# Complete Record of the Surgery of the Battles Fought near Vicksburg, Dec. 27, 28, 29, and 30, 1862

**Published:** 1863-01

**Authors:** E. Andrews

**Affiliations:** Late Surgeon of 1st Reg. Ill. Light Artillery, and Professor of Surgery in the Medical Department of Lind University


ARTICLE II.

### COMPLETE RECORD OF THE SURGERY OF THE BATTLES FOUGHT NEAR VICKSBURG, DEC. 27, 28, 29, and 30, 1862.

By E. ANDREWS, late Surgeon of 1st Reg. Ill. Light Artillery, and Professor of Surgery in the Medical Department of Lind University.

A complete record of the surgery of any battle during the present war, is a thing which, heretofore, has seldom been attempted.

Both in the east and the west, the urgency of military movements, and the confusion of battle, have made futile the imperfect attempts at registration adopted, and the vast statistics of the war have slipped forever from our hands.

In the west, the wounded have usually been taken from the Field Hospitals to the Hospital Boats, and by them taken on long river voyages to General Hospitals in our cities. The operations and deaths were not communicated by the field-surgeons to the surgeons of the boats, and the surgery and mortality on the boats were not faithfully furnished to the General Hospitals. The statistics of the Field, the Boats, and the General Hospitals, therefore, are not combined, and no continuous history of the cases can be traced. In this, and in similar ways, have the enormous statistics of almost all our great battles been lost to the profession, and the vast and costly experience of so much blood and death been rendered worthless for the settlement of the many difficult questions in practical surgery.

It was with intense chagrin that I thus saw the entire loss of scientific results from the bloody battles of Fort Donelson, Shiloh, and the numerous lesser combats in front of Corinth. It is a painful fact, that after these battles the results of the various operations and injuries remained entirely unknown to the original operators, and they gained almost nothing by their experience, except the skill of hand acquired in their manipulations.

For this reason, I resolved at the next large battle in which I should be engaged, to make a determined effort to secure the entire surgical history of the wounded up to the latest period which the circumstances would permit. In this endeavor I have been successful. Owing to the judicious orders of Medical Director, Dr. CHARLES McMILLAN, the field records were measurably well kept, and by the help of Dr. H. B. WITT, senior assistant-surgeon of the 69th Indiana Infantry, who entered into my plans with great energy, I have been put in possession of the subsequent history of the cases, for the most part, up to the twentieth day after the battle.

Dr. WITT was with the wounded personally up to that time, and displayed great skill and capacity in his operations and management.

I am also indebted to the assistance of Dr. TURNER, the well-known surgeon of the hospital steamer "City of Memphis," for valuable information in completing the record.

The following order will show the arrangements adopted to secure order and efficiency on the field:—

CIRCULAR.

HEAD-QUARTERS RIGHT WING 13TH ARMY CORPS,
ON BOARD STEAMER "FOREST QUEEN,"
*December 20th*, 1862.

To the end that the Medical Staff of this command may act with the greatest possible efficiency, in the necessary and proper care and treatment of the wounded on the battle field, the following instructions are issued for the guidance of Regimental and Division-Surgeons:—

The present organization of the division gives but one principal medical officer, who is attached to the staff of the general commanding, and upon whom devolves the administrative duties. All other surgeons are relieved from duty with brigades, and will, therefore, be charged only with the care of their own regiments.

Before a battle, the senior surgeons of divisions will select a proper and convenient place to serve as a principal depot and field-hospital; notifying the surgeons of his division and the medical director of its location; and will make such arrangements as shall secure the prompt delivery, by the litter-bearers and ambulances of his division, of the wounded of the command, in order that they may receive immediate attention.

To secure the prompt delivery at the depot, and the immediate necessary attention, the hospital service will be systematized as follows:—

Division-surgeons will direct all ambulances belonging to the division to report to them at once, so soon as an action is deemed imminent, and will proceed to fit up their depot, asking for that service, for a sufficient detail under the charge of a competent lieutenant from the division commander. This detail should be made from the regiments, and should be large enough to furnish two men to each ambulance, in addition to the driver, who should not leave his team. These should not be boys and worthless old men; but strong, brave, and efficient ones. They will be distinguished by a strip of white bandage tied around the arm above the elbow; and no others shall be permitted to leave the ranks and carry wounded to the rear. The bands will assist in pitching tents and preparing shelter, and fuel, fires, and nourishment for the wounded. And these, with the above-mentioned detail, shall be placed under the charge of an assistant-surgeon, who shall be selected for the superintendence of this department of hospital duty.

Furthermore, the Hospital shall be organized as follows:—

Three principal operators shall be selected from the Medical

Staff of the Division-Surgeon; and they, under the direction of this senior medical officer, shall decide upon and perform all principal operations. They will be selected without reference to rank, but solely for the requisite qualifications and experience. Each operator shall have one assistant, to be selected in the same manner. One efficient assistant shall be selected to keep the records of the depot, and another to attend as above mentioned, to the providing of food, shelter, &c. It is understood that one assistant-surgeon with his hospital steward and attendants, shall accompany the regiment to which he is attached to the field, and select and station himself at a convenient and safe place in the rear, to which the wounded may be first brought from the ranks, where temporary dressings may be applied, and where the ambulances may collect them for transmission to the hospital or principal depot. He should be relieved, if the action continues, by another, that justice be done to all and each. All the medical officers should immediately report at the principal depot of their division, and assist in the general care of the wounded.

The division trains being usually posted in a secure place, and at generally too great a distance to make resort to the regimental medical stores in the wagons available; the medicine wagons, pannier sets, and hospital knapsacks, should be reserved with the ambulances before the commencement of an action, from the Quarter-Master's train, and used for the occasion as necessity may require. The knapsacks, as above mentioned, and the medicine wagons and pannier sets, with the proper proportion of instruments to be placed under the orders of the surgeon in charge at the principal depot. Care should be taken that the supplies of chloroform, ether, and stimulants are present and available.

Beef should be obtained at once, and with the stores of farina, tea, &c., the wounded can at once be nourished and made comfortable. Such cooks as shall be selected, shall be ordered to the principal depot; and such attendants as are not needed by the surgeon in the field, will assist in the care and nursing of the wounded.

Prompt and careful compliance with these instructions, it is hoped, will secure to our brave officers and soldiers who may be wounded in the battles which may follow, such care and treatment as they nobly deserve; and such as their much sacrificing friends at home have just right to expect.

(Signed.) CHAS. McMILLAN,
Medical Director, Right Wing 13th Army Corps.

By this arrangement it will be seen that one assistant-surgeon accompanied each regiment under fire, to attend to such injuries as might require instantaneous attention. The wounded were thence taken to division depots about 200 yards from the line of battle, for full examination, and at these depots all principal operations were attended to. Three surgeons were appointed operators in each division, and by them all serious operations were performed. One assistant-surgeon was appointed at each depot to make record of the name, company, regiment, injury, operation, and name of the operator of each patient brought in.

Being appointed one of the operators, I had opportunity to know that the recorder of my division, (Dr. Brown, 113th Ill. Infantry,) was very careful and thorough in his notes, and I now have his original field-registar before me for my guidance. The following order shows the arrangements adopted in the 2d division, which was substantially like those in the others:—

CIRCULAR.

Head-Quarters 2d Division, Right Wing 13th Army Corps,
On Board Steamer "Chancellor,"
*December 25th*, 1862.

In accordance with Circular bearing date December 20th, 1862, the following named medical officers have been selected, and will act in the particular position here assigned to them in case of a battle. The following named medical officers are selected as principal operators at division hospital:—

| | | |
|---|---|---|
| E. Andrews, | Surgeon, . | 1st Ill. Light Artillery. |
| E. O. F. Roler, | " . | 54th " Infantry. |
| G. S. Walker, | " . | 6th Mo. " |

For assistants to same:—

| | | |
|---|---|---|
| D. W. Carlin, | Surgeon, . | 57th Ohio. |
| J. R. Bailey, | " . | 8th Mo. |
| C. P. Brent, | " . | 54th Ohio. |

Recorder for Division Hospital:—

L. C. Brown, 1st Assistant-Surgeon, 113th Ill.

To take charge bands, cooks, &c., for preparing food, shelter, and fuel at division hospital:—

H. C. Vinsen, 2d Assistant-Surgeon, 83d Ind.

The following Medical Officers will report at division hospital for duty:—

| | | |
|---|---|---|
| I. N. Heckalmann, | 1st Assistant, | 116th Ill. |
| L. Davis, | Surgeon, . . | 83d Ind. |
| J. R. Gore, | " . . | 127th Ill. |
| Wm. Turner, | Assistant-Surgeon, | 1st Ill. Artillery. |
| James M. Mack, | Surgeon, . | 113th Ill. |

The following named Medical Officers will accompany their regiments into action, each having under his charge the hospital stewards and all other hospital attendants, except these that may be detailed for duty at the division hospital. The hospital steward will carry the knapsack filled with such articles as may be necessary for immediate use. Medical officers will give their personal attention, and see that their medical supplies are at division hospital:—

| | | |
|---|---|---|
| E. M. Joslin, | 1st Assistant-Surgeon, . | 6th Mo. |
| Ivlus Brown, | " . | 8th " |
| J. Baygo, | . . . . . . | 54th Ohio. |
| S. L. Harper, | . . . . . . | 55th Ill. |
| A. C. Messenger, | . . . . . | 57th Ohio. |
| W. Gillispi, | . . . . . . | 83d Ind. |
| W. N. Bailey, | . . . . . . | 113th Ill. |
| J. A. W. Hartiller, | . . . . . . | 116th Ill. |
| G. P. Anthony, | . . . . . . | 127th " |
| I. Huss, | . . . . . . . | 13th U. S. A. |

D. W. HARTSHORN,
Medical Director, 2d Division.

The ambulances were worked in two sections: one portion bringing the wounded from the front to the depot, and the other taking them away, after the wounds were dressed, to the hospital boats.

In this way the wounded were all attended to without confusion, and most of the time without haste, and with few exceptions, the injured of each day were safely lodged on the hospital boats the same night. I must do the operators also the justice to say that they performed their duty well, avoiding with good judgment the two extremes of reckless slashing and dangerous ultra conservatism. A few of the assistant-surgeons who were sent under fire, became so exhilerated at the music of the bullets, as to expose themselves to an unnecessary amount of danger, but not a man of them proved cowardly.

After the battle, the fleet left for the mouth of White River, Arkansas; and from thence the wounded were taken to Memphis, afterwards to St. Louis. In all these movements about twenty days were consumed, which is sufficient to show the probable results of the operations. My record closes in most cases with the nineteenth and twentieth days after the battle, when the cases were turned over to General Hospital in St. Louis. The following table contains the particular cases and results, and therefore, are the basis and proof of the remarks, and conclusions following them; and it is peculiarly gratifying to me that at length we are able to bring the maxims of military surgery to the corrective test of a large collection of facts, obtained on the western fields:—

Tabular View of the Wounds and Surgical Operations of the late Battles near Vicksburg, on the 27th, 28th, and 29th of December, 1862, with Addenda from other Western Battles.

WOUNDS OF THE HEAD.

| Case. | Name. | Regiment. | Injury. | Operation. | Anæsthetic. | REMARKS. |
|---|---|---|---|---|---|---|
| 1 | N. A. | 49 Indiana | Shot in mouth | Ball not extracted | None | Very weak 15th day |
| 2 | G. B. | 16 Ohio | Shell wound of back of head, right side | None | | Left leg paralyzed |
| 3 | S. M. | 54 Indiana | Shell wound, left eye and temple | " | | Had erysipelas,—better |
| | | | | | | Doing well 15th day |
| 4 | J. H. | 6 Missouri | Eye injured | " | | do do |
| 5 | S. B. M. | 54 Indiana | Severe scalp wound, right side | " | | Worse 16th day |
| 6 | W. J. | 16 Ohio | Scalp wound, left side head | " | | Doing well 16th day |
| 7 | A. H. | 6 Missouri | do top of head | " | | do |
| 8 | H. C. | 83 Indiana | do back of head | " | | do |
| 9 | J. S. | 4 Iowa | Bullet entered left side of mouth | Bullet not found | | do |
| 10 | J. R. | 6 Missouri | Scalp wound, top of head | None | | do |
| 11 | W. F. | 42 Ohio | Shell wound of forehead | " | | do 17th day |
| 12 | J. W. | do | Contusion of temple | " | | do |
| 13 | W. W. F. | 54 Indiana | Bullet enter'd below right ear, passed out back of neck | " | | do |
| 14 | W. W. R. | 16 Ohio | Bullet pierced nose, shattered palate bone, and passed into region of fauces | Bullet extracted | | do 18th day |
| 15 | S. R. | do | Scalp wound, top of head | None | | Erysipelas, doing well 18th |
| 16 | G. V. S. | 54 Indiana | Ball enter'd mouth, knock'd in front teeth | Bullet not extracted | | Secondary hemorrhage 11th day. Check'd with persulph. iron; doing well 17th day |
| 17 | A. D. | do | General concussion by shell | None | | Doing poorly 17th day |
| 18 | T. B. | 29 Missouri | Shell wound, destroyed right eye | " | | Doing well 18th day |
| 19 | W. B. A. | 30 Iowa | Shell wound, side of head | " | | |
| 20 | A. W. B. | 4 do | Ball entered mouth, passed out under left | | | Not doing well 18th day |
| | | | ear | " | | Doing well 18th day |

| Case. | Name. | Regiment. | Injury. | Operation. | Anæsthetic. | Remarks. |
|---|---|---|---|---|---|---|
| 21 | E. A. G. | 57 Ohio | Shell wound, right temple | None | | Doing well 20th day |
| 22 | M. B. | 114 do | Compound fracture of skull | " | | Died 12th day |
| 23 | J. H. E. | 54 Indiana | Shot in face | " | | do 7th day |
| 24 | McE. K. | 49 do | do cranium | " | | do 1st do |
| 25 | J. H. | 16 Ohio | do do | " | | do |
| 26 | E. S. | do | do do | " | | do 13th day |
| 27 | P. S. | 83 Indiana | Wound of ear | " | | |
| 28 | J. H. | do | Comp. fracture frontal bone, dura mater not opened | Removed loose fragm'ts bone | None | |
| 29 | W. W. | 6 Missouri | Shell wound, right temple | None | | |
| 30 | W. McK. | 54 Ohio | Bullet entered brain through right eye | " | | |
| 31 | J. S. | 13 Regulars | Scalp wound | " | | |
| 32 | M. A. | 6 Missouri | Compound fracture of lower jaw | " | | |
| 33 | G. C. | 16 Ohio | Slight scalp wound | " | | Returned to duty |
| 34 | S. W. | do | do wound of cheek | " | | Doing well 10th day |
| 35 | C. W. J. | 54 Indiana | do do | " | | do |
| 36 | P. B. | do | Scalp wound | " | | do 8th day |

*Addenda from notes of other Western Battles.*

| | | | | | | |
|---|---|---|---|---|---|---|
| 37 | J. J. | 8 Iowa | Comp. fr. of skull. Deep depression; compression of brain | Trepanned 9th day | None | Had erysipelas; died 10th day |
| 38 | F. A. | 17 Illinois | Shot in sup. max. bone | None | | Exfoliation of bone, recover'd. |
| 39 | B. C. | 6 Ill. Cav. | 6 buck-shot in face, one penetrating brain | " | | Died 5th day |

To the above should be added eleven slightly wounded cases, who were returned to their Regiments. Total, 50.

### WOUNDS OF THE NECK, TRUNK, AND SHOULDER.

| | | | | | | |
|---|---|---|---|---|---|---|
| 1 | C. S. | 22 K'ntucky | Flesh wound, abdominal wall | None | None | Doing well 20th day |
| 2 | J. H. D. | do | Upper lobe of left lung | " | " | do |
| 3 | C. M. | 16 Ohio | Shot thro' breast, above and internal to nipple, ball came out the back | " | " | do |
| 4 | J. D. H. | do | Flesh wound of shoulder | " | " | Doing badly 15th day |
| 5 | S. S. | 49 Indiana | do | " | " | Very weak do |
| 6 | B. F. M. | do | Bullet through left chest | " | " | Doing well do |
| 7 | T. W. S. | 16 Ohio | do left lung, air escaped | Ball cut out below scapula | | do |
| 8 | H. A. D. | 114 Ohio | Shell wound of back | None | | do |
| 9 | J. P. L. | 54 Indiana | do and hip | " | | Parts below paralyzed |
| 10 | J. H. H. | do | do left side of neck | " | | Doing toler'y well 15th day |
| 11 | A. B. | 16 Ohio | Ball entered left side of neck, below ear, and escaped over rt scapula | " | | Doing well 16th day |
| 12 | G. S. | 42 do | Shell wound of back | " | | Doing toler'y well 15th day |
| 13 | J. E. | 29 Missouri | Ball passed thro' right chest and arm | " | | Doing well 15th day |
| 14 | D. P. V. | 54 Indiana | Flesh wound left side of neck | " | | do |
| 15 | B. F. S. | 13 Illinois | do shoulder | Ball not extracted | | Doing toler'y well 15th day |
| 16 | W. S. | 42 Ohio | Contusion of back | None | | No better 16th day |
| 17 | H. S. | 54 Indiana | do breast by shell | " | | Pain in breast 16th day |
| 18 | G. S. | 31 Missouri | Flesh wound, left shoulder | " | | Doing well 16th day |
| 19 | R. D. | 16 Ohio | Wound, penetrating left side | " | | Doing badly do |
| 20 | H. M. | 49 Indiana | Flesh wound, shoulder | " | | Doing well do |
| 21 | A. B. | do | do side of neck | " | | do |
| 22 | G. C | 22 K'ntucky | do right shoulder | " | | do |
| 23 | J. W. | 25 Iowa | Bayonet wound of shoulder | " | | do |
| 24 | H. L. | 22 K'ntucky | Ball passed across front of neck, anterior to arteries | " | | do |
| 25 | R. V. L. | 16 Ohio | Flesh wound, ball glided around on rib of left side, beneath the skin | " | | do 17th day |
| 26 | M. B. | 7 Mich. Bat. | Flesh wound of back, over 6th rib | Bullet cut out | | do |
| 27 | J. McD. | do | do abdomen, by cannister, not penetrating cavity | Iron shot cut out on 9th day | | do |
| 28 | C. W. H. | 31 Missouri | Flesh wound, right shoulder | None | | do |

| Case. | Name. | Regiment. | Injury. | Operation | Anæsthetic. | Remarks. |
|---|---|---|---|---|---|---|
| 29 | H. S. | 29 Missouri | Flesh wound, right shoulder | None | | Doing well 18th day |
| 30 | A. Y. | 28 La. | Bullet entered near shoulder | Cut out near spine | | Erysipelas on 17th day |
| 31 | J. P. | 13 Missouri | Right lung penetrated | None | | Doing well 20th day |
| 32 | R. W. | 4 Iowa | Flesh wound, left side | Ball cut out from back | | do 18th day |
| 33 | J. M. | 6 Missouri | do both shoulders | None | | do |
| 34 | S. C. | 13 do | do right shoulder | Ball extracted | | do |
| 35 | H. R. | 6 do | Shot through left breast | " at left scapula | None | Not doing well 18th day |
| 36 | O. C. | 29 do | do right breast | None | | Doing well 18th day |
| 37 | J. J. | 54 Ohio | do lung | " | | do |
| 38 | F. T. | 29 Missouri | do lower end of spine | " | | do |
| 39 | J. E. | do | Flesh wound, left shoulder | " | | do |
| 40 | C. G. | 13 Illinois | Ball entered near shoulder and passed out by 12th rib | " | | do |
| 41 | O. P. W. | do | Shot through right upper lung | " | | do |
| 42 | J. S. | do | Fracture of right shoulder | " | | do |
| 43 | H. G. | do | Ball entered near scapula | Ball cut out | | do |
| 44 | F. Z. | 31 Missouri | Flesh wound, trunk | None | | do |
| 45 | M. M. | 6 do | do | " | | Not doing well 18th day |
| 46 | A. D. W. | 13 Illinois | Flesh wound, back | " | | Doing well 18th day |
| 47 | S. A. D. | 4 Iowa | do left shoulder | " | | do |
| 48 | M. C. | 13 Regulars | do back | Ball extracted | None | do 20th day |
| 49 | F. L. M. | 4 Iowa | do side and hip | None | | do |
| 50 | F. S. | 6 Missouri | do side and back | " | | do |
| 51 | E. E. A. | 31 do | do neck and shoulder | " | | do |
| 52 | W. F. C. | 55 Illinois | do shoulder | " | | do |
| 53 | A. B. C. | 42 Ohio | Shot in left shoulder | " | | Died 7th day |
| 54 | J. K. | 54 Indiana | do head, breast, and leg | " | | do 1st day |
| 55 | G. H. | 16 Ohio | Flesh wound, right shoulder | " | | |
| 56 | C. F. | 28 La. | Shot through right breast | " | | Rebel, died 12th day of pneu. |
| 57 | W. R. | 49 Indiana | Penetrating wound in side | " | | Died 7th day |
| 58 | A. W. C. | 42 Ohio | do breast | " | | do 6th day |
| 59 | B. B. D. | 40 Indiana | do abdomen | " | | do 1st day |

| | | | | | | |
|---|---|---|---|---|---|---|
| 60 | W. M. | 1 Wis. Bat. | Penetrating wound in abdomen | " | | do |
| 61 | J. C. H. | 22 K'ntucky | Body and arm | " | | do 12th day |
| 62 | M. R. | 54 Indiana | Right lung shot through | " | | do 8th day |
| 63 | C. B. | 13 Illinois | Shot in the right lung | " | | do |
| 64 | J. T. | 22 K'ntucky | Ball entered chest, through left shoulder | " | | do 6th day |
| 65 | P. W. E. | 16 Ohio | Ball entered side, through arm | " | | do |
| 66 | A. J. | 54 Indiana | Shot in breast | " | | do 12th day |
| 67 | R. P. | 22 K'ntucky | do right breast | " | | do 5th day |
| 68 | A. M. | 54 Indiana | do shoulder and foot | " | | do 9th day |
| 69 | T. M. | 114 Ohio | do side and back | " | | do |
| 70 | R. S. | 3 Kentucky | do side | " | | do 12th day |
| 71 | S. H. | 57 Ohio | Flesh wound, neck | " | | |
| 72 | G. R. R. | 83 Indiana | Shot through left shoulder | " | | |
| 73 | E. W. | do | Bullet penetrating left lung | " | | Died 1st day |
| 74 | J. B. | 127 Illinois | Flesh wound, right shoulder | " | | |
| 75 | F. H. | 54 Ohio | Contusion of epigastrium by shell | " | | |
| 76 | C. R. | do | General concussion by shell | " | | |
| 77 | W. H. | do | do | " | | |
| 78 | E. V. | do | do | " | | |
| 79 | J. D. P. | do | Contusion of body by shell | " | | |
| 80 | R. L. V. | 6 Missouri | Ball penetrated abdomen near naval | " | | |
| 81 | R. W. | 54 Ohio | Contusion from spent ball on chest | " | | Died |
| 82 | J. S. | 13 Regulars | Bullet pierced right lung | Ball extracted | | |
| 83 | J. S. | 6 Missouri | do flesh of back | " cut out at shoulder | None | |
| 84 | A. B. | 13 Regulars | do pierced right lung | " " " of back | " | |
| 85 | Y. F. D. | 6 Missouri | Contusion of back from shell | None | " | |
| 86 | M. C. | do | Flesh wound, back of neck | Ball extracted | | |
| 87 | J. F. | do | Bullet entered back and pierced left lung | " not extracted | | |
| 88 | M. C. | do | Flesh wound, pectoral muscles | None | | |
| 89 | Col. B. | do | Contusion, left shoulder | " | | |
| 90 | T. S. | do | Bullet pierced right lung | " | | |
| 91 | J. D. | do | Ball entered back and pierced left lung | " | | Died |
| 92 | M. McN. | do | Shot in left lung | " | | |
| 93 | J. M. | do | do abdomen | " | | |

| Case. | Name. | Regiment. | Injury. | Operation. | Anæsthetic. | Remarks. |
|---|---|---|---|---|---|---|
| 94 | H. H. | do | Flesh wound in back | " | | |
| 95 | E. Z. | do | do right shoulder | " | | |
| 96 | J. S. | do | Shot in abdomen | " | | Died |
| 97 | W. S. | 22 K'ntucky | Contusion of shoulder | " | | Returned to duty |
| 98 | B. S | 16 Ohio | General concussion | " | | do |
| 99 | W. A. | 54 Indiana | do | " | | Nearly well 8th day |
| 100 | J. D. | do | do | " | | Returned to duty |
| 101 | M. C. H. | do | do | " | | do |
| 102 | R. M. C. | 42 Ohio | do | " | | do |
| 103 | J. S. | 120 do | do | " | | do |
| 104 | A. J. | do | do | " | | do |
| 105 | E. B. | 13 Illinois | Shell wound, perforating intestine | " | | Died 8th day |

*Addenda from notes of other Western Battles.*

| Case. | Name. | Regiment. | Injury. | Operation. | Anæsthetic. | Remarks. |
|---|---|---|---|---|---|---|
| 106 | J. C. | 8 Missouri | Shot thro' abdomen, piercing colon | Bullet cut out from back | None | Died 4th day of peritonitis |
| 107 | M. W. | 6 do | Large piece of shell in lumber muscle | Extracted | " | Result unknown |
| 108 | C. H. C. | do | Bullet passed thro' right arm into thorax | None | | do |
| 109 | S. S. | 4 Ind. Bat. | Shot thro' left shoulder, frac. of clavicle | Extracted fragments | Chlor. | Doing well 14th day |
| 110 | J. H. | Servant | Stab'd in abdomen, cutting small intestine | Intestines sewed up with interrupted sutures & return'd | | Recovered readily; had hernia some months after |
| 111 | J. B. | 40 Illinois | Bullet pierced thorax | Extracted | Chlor. | Recovered |
| 112 | B. D. | do | do fracture of 7th rib | Resected rib | " | do |
| 113 | A. A. | 46 do | Compound fracture of scapula | Extracted fragments | " | do |
| 114 | — D. | 40 do | Ball enter'd r. thorax, ext'nsive emphysema | Tapped | None | do |
| 115 | S. S. | do | Shot in left shoulder, and frac. of 1st rib | None | | do |
| 116 | O. E. | do | Comp. fracture of scapula,—grapeshot | " | | Unknown |
| 117 | S. H. | Unknown | do head of humerus, thorax pierc'd | " | | Died 9th day of 2d hæmorr. |
| 118 | M. L. | 41 Illinois | Flesh wound of neck | " | | Recovered |
| 119 | J. B. | 6 do | Stab'd in abdomen, viscera not wounded | " | | do |
| 120 | R. N. | Servant | Stabbed, cutting subscapular artery | Cut down and tied | Chlor. | do |

| | | | | | | |
|---|---|---|---|---|---|---|
| 121 | A. B. | Contraband | Pistol-shot in lung, by his master | None | | Died 10th day |
| 122 | C. C. | do | Shot in shoulder, by his master | do | | Recovered |
| 123 | C. S. | do | do hip do | Extracted ball | | do |
| 124 | S . | 6 Ill. Cav | Shot in arm and breast, flesh wound | None | | do |
| 125 | —— | do | Flesh wound in breast by buckshot | do | | do |
| 126 | —— | do | do side do | Extracted | | do |
| 127 | —— | do | do do do | do | | do |

2A

With these should be reckoned forty-seven slight cases wounded at Vicksburg, who were not disabled, and remained with their Regiments. Total wounds of Neck, Trunk, and Shoulder, 174.

### WOUNDS OF ARM.

| | | | | | | |
|---|---|---|---|---|---|---|
| 1 | C. S. | 42 Ohio | Fracture of head of humerus | Resection on the field | Chlor. | Doing well 20th day |
| 2 | W.W.W. | 54 Indiana | Flesh wound near elbow | None | None | Has fever 20th day |
| 3 | C. G. | 16 Ohio | do right arm | do | do | Tolerably well 15th day |
| 4 | G. C. | 30 Missouri | Shell wound of arm, very bad comp. frac. | Primary amputation | Chlor. | Doing well 15th day |
| 5 | J.C.McC. | 49 Indiana | Compound fracture head humerus | None | | do |
| 6 | W. F. P. | 69 do | Ball entered top shoulder, passed 4 inches down arm | Ball extracted | | do<br>do do |
| 7 | J. E. | 22 Kentucky | Compound fracture, left arm | None | | do do |
| 8 | R. W. | 54 Indiana | Bad flesh wound, right arm | do | | do do |
| 9 | E. F. T. | do | Flesh wound | do | | Doing well 16th day |
| 10 | B. B. | 16 Ohio | do both arms | do | | do do |
| 11 | F. C. M. | 13 Illinois | do left arm | do | | |
| 12 | C. A. B. | do | do right arm | do | | |
| 13 | M. G. | 58 Ohio | do do | do | | |
| 14 | W. M. B. | 22 Kentucky | do do | do | | |
| 15 | C. G. | 16 Ohio | do arm | do | | |
| 16 | S. S. J. | 54 Indiana | Compound fracture, humerus | Secondary amput. at upper 3d | do | do 17th day |
| 17 | E.O.G.R. | 16 Ohio | Flesh wound, middle of arm | None | | do 18th day |
| 18 | H. J. R. | 114 do | Flesh wound, ball enter'd arm and pass'd out at shoulder | do | | |
| 19 | P. H. | 16 do | Flesh wound, upper part of arm, injuring artery. 2d hæmor. & gangrene 11th day | Ligated subclavian 11th day, amputated arm | do | Recovered |

| Case. | Name. | Regiment. | Injury. | Operation | Anæsthetic. | Remarks. |
|---|---|---|---|---|---|---|
| 20 | W. S. | 127 Illinois | Compound fracture head of left humerus | Exsection on field | Chlor | Doing well 18th day |
| 21 | W. A. | 58 Ohio | do do of humerus | Primary amp. shoulder-joint | do | do do |
| 22 | A. H. T. | 4 Iowa | do do do | Resection of shoulder | do | do do |
| 23 | W. J. C. | do | Flesh wound left arm and breast | None | | do do |
| 24 | Z. B. F. | 13 Illinois | Compound fracture right humerus | do | | do do |
| 25 | J. S. | do | do do do | do | | do do |
| 26 | R. A. L. | 83 Indiana | do do left humerus & radius | Bullet and pieces of bone cut out by deltoid muscle | do | do do |
| 27 | J. A. H. | 13 Illinois | do do humerus | Amputation at shoulder-joint | do | do do |
| 28 | G. W. D. | 4 Iowa | do do do | High amputation on field | do | do do |
| 29 | D. A. C. | 29 Missouri | do do do | Amp. near shoulder on field | do | do do |
| 30 | L. H. E. | 6 do | do do do | Shoulder resected on field | do | do do |
| 31 | S. G. | 13 Illinois | do do do by shell | Primary amputation | do | do do |
| 32 | W. B. | 55 do | do do do bad | None | | do 20th day |
| 33 | H. V. | 22 Kentucky | do do do | Amputation at shoulder-joint | do | Died 9th day |
| 34 | J. P. | do | do do right arm | None | | do 7th do |
| 35 | G. W. F. | 57 Ohio | do do do by shell | Primary amp. at lower 3d | do | |
| 36 | W. B. | 55 Illinois | do do humerus | Bullet cut out | do | |
| 37 | S. B. C. | 13 Regulars | do do do | Resection of shoulder | do | |
| 38 | — I. | 6 Missouri | Flesh wound right arm | None | | |
| 39 | J. H. | 54 Indiana | do do slight | do | | Nearly well 12th day |
| 40 | E. B. P. | do | Compound fracture arm | Primary amputation | do | Doing well 6th day |
| 41 | J. R. | do | do do do | do do | do | do do |
| 42 | H. C. B. | 16 Ohio | Flesh wound arm | None | | do do |
| 43 | J. J. | Unknown | Compound fracture humerus | Amputation shoulder-joint | do | Doing well 28th day |

*Addenda from notes of other Western Battles.*

| Case. | Name. | Regiment. | Injury. | Operation | Anæsthetic. | Remarks. |
|---|---|---|---|---|---|---|
| 44 | C. V. | do | do do do | do do | do | do 15th day |
| 45 | C. S. | do | do do do | do do | do | Died 4th day |
| 46 | S. S. | do | Arm torn off at the shoulder by shell | No amputation, artery tied | None | do |
| 47 | S. W. | 8 Missouri | Comp. fracture head of humerus & lo. jaw | Resection of shoulder-joint | Chlor | Recovered |

| | | | | | | |
|---|---|---|---|---|---|---|
| 48 | C. C. M. | 40 Illinois | Flesh wound right arm | None | | do |
| 49 | S. S. | 6 Ill. Cav. | do do do | Extracted buckshot | None | do |
| 50 | H. R. | do | do do do | od do | do | do |

With the above should be reckoned 18 slight wounds of the arm, from the Vicksburg Battles, which remained with their Regiments and all recovered. Total injuries of the Arm, 69.

### WOUNDS OF ELBOW.

| | | | | | | |
|---|---|---|---|---|---|---|
| 1 | J. I. | 42 Ohio | Left elbow-joint opened | None | —— | Arm cons'ly swollen 15th day |
| 2 | J.McH. | 49 Indiana | Flesh wound elbow | do | | Doing well 16th day |
| 3 | T. G. C. | 54 do | Contusion from cannon ball | do | | do do |
| 4 | F. E. L. | 7 Mich. Bat. | Shell wound right elbow | do | | Erysipelas, better on17th day |
| 5 | J. H. | 13 Illinois | Bullet through right elbow | Amputation of arm on field | Chlor. | Doing well 18th day |
| 6 | M. H. | do | Comp. frac., right elbow | None | | do do |
| 7 | F. M. S. | 6 Missouri | do also flesh w'd. in neck | Resected elbow-joint | do | do do |
| 8 | J. H. | 83 Indiana | Comp. fracture elbow-joint | None | | |
| 9 | E. A. | 116 Illinois | Comp. fracture left elbow-joint | Amputation of arm | do | |
| 10 | P. H. | 16 Ohio | do right do | do do | do | do 6th day |

### *Addenda from notes of other Western Battles.*

| | | | | | | |
|---|---|---|---|---|---|---|
| 11 | D. D. D. | 8 Missouri | Comp. fracture right elbow-joint | Excision of joint | do | Recovered |
| 12 | E. B. | 55 Illinois | do do do | do do | do | Doing well 16th day |
| 13 | W. M. | Unknown | do do do | Joint excised 8th day | do | Died same day |
| 14 | G. W. | 40 Illinois | do left do | Extracted fragments | do | Recovered, on duty |

Total injuries of the Elbow, 14.

### WOUNDS OF FORE-ARM.

| | | | | | | |
|---|---|---|---|---|---|---|
| 1 | | | Flesh wound ball lodged between radius and ulna near elbow | Extracted 12th day | Un'kn | Had erysipelas doing better prospect good on 20th day |
| 2 | G.W. H. | 16 Ohio | Flesh wound | None | None | Doing well 15th day |
| 3 | A. R. | do | do | Ball not extracted | do | Erysipelas on 15th day |
| 4 | S. J. H | 49 Indiana | Bullet passed between radius and ulna | None | do | Tolerably well on 15th day |
| 5 | C. F. | 16 Ohio | Compound fracture of ulna | do | | Doing well 16th day |
| 6 | J. T. B. | 42 Ohio | Flesh wound, ball entered near wrist and passed out above elbow | do | | do do |

| Case. | Name. | Regiment. | Injury. | Operation. | Anæsthetic. | Remarks. |
|---|---|---|---|---|---|---|
| 7 | D. T. D. | 22 K'ntucky | Flesh wound right fore-arm | None | | Fever on 16th day |
| 8 | H. A. B. | do | do left fore-arm | do | | Doing well 16th day |
| 9 | S. S. | 16 Ohio | do do | do | | do do |
| 10 | A. S. | 29 Missouri | do right fore-arm | do | | do do |
| 11 | A. S. D. | 22 K'ntucky | Compound fracture, both bones | do | | Doing well 17th day |
| 12 | E. B. P. | 54 Indiana | do do by grape | Amputated lower 3d of arm | Chlor. | do do |
| 13 | J. R. | 42 Ohio | Ball lodged in bone | None | | Erysipelas 17th day |
| 14 | L. R. | 31 Missouri | Flesh wound left fore-arm | Bullet not extracted | | Doing well 18th day |
| 15 | J. P. | 16 Ohio | do right do | None | | do do |
| 16 | W. W. J. | 31 Missouri | Compound fracture right fore-arm | do | | do do |
| 17 | H. M. B. | 29 do | Bullet passed between bones | do | | do do |
| 18 | J. J. M. | 31 do | Flesh wound fore-arm | do | | Had erysipelas on face, doing well 16th day |
| 19 | J. W. | do | do right fore-arm | do | | Doing well 18th day |
| 20 | E. E. | 116 Illinois | Compound fracture do | Amputation on field | Chlor. | do do |
| 21 | H. W. B. | 29 Missouri | do do left fore-arm | None | | Not doing well 18th day |
| 22 | C. M. | 6 do | Shell fractured radius | do | | Doing well 18th day |
| 23 | M. S. | 9 Iowa | Flesh wound right fore-arm | do | | do do |
| 24 | R. A. S. | 4 do | do left do | do | | do 20th day |
| 25 | R. A. S. | 13 Illinois | Compound fracture middle of fore-arm | Primary amputation | " | do do |
| 26 | G. O. | 83 Indiana | do left ulna | None | | |
| 27 | C. I. | 6 Missouri | do ulna by shell | Extracted piece of shell and fragment of bone | None | |
| 28 | R. G. | do | Flesh wound right fore-arm | Bullet extracted | " | |
| 29 | B. A. | do | do left do | None | | |
| 30 | A. S. | 69 Indiana | Compound fracture fore-arm | do | | |

*Addenda from other Western Battles.*

| | | | | | | |
|---|---|---|---|---|---|---|
| 31 | J. H. | 1st Ill. Art. | Fore-arm blown off | Amputation, middle fore-arm | Chlor. | Recovered |
| 32 | J. M. | 40 Illinois | Compound fracture, left radius | None | | do |
| 33 | J. B. | 29 do | do | Extracted fragments | " | do |

| | | | | | | |
|---|---|---|---|---|---|---|
| 34 | J. M. | 40 Illinois | Flesh wound, left fore-arm | None | | do |
| 35 | —— | 6 Ill. Cav. | Buckshot in right fore-arm | Extracted | | do |

With the above should be reckoned 8 cases of slight wounds of fore-arm, received in the Vicksburg fights, which remained with their regiments. Total wounds of fore-arm, 43.

WOUNDS OF HAND.

| | | | | | | |
|---|---|---|---|---|---|---|
| 1 | Z. M. | 34 Indiana | Flesh wound, hand | None | None | Doing well 15th day |
| 2 | H. A. D. | 114 Ohio | Bullet through hand | do | do | do do |
| 3 | H. C. B. | 16 Ohio | Compound fracture of wrist by shell | do | do | Doing toler'y well 15th day |
| 4 | G. D. C. | 54 Indiana | Ball fract'd 2d finger right hand, entered centre left hand, pas'd out above wrist | Ball cut out above left wrist; finger amputated | Chlor. | Doing well 15th day |
| 5 | G. S. | 42 Ohio | Flesh wound, fingers | None | | do do |
| 6 | W. G. | 114 Ohio | Fracture of index finger | Amputated | do | do do |
| 7 | D. C. H. | 69 Indiana | Flesh wound, left index finger | None | | do do |
| 8 | E. R. C. | do | Comp. fracture of all fingers of 1 hand | Amputation of all 4 fingers | do | Hand ulcerated 15th day |
| 9 | W. S. | 42 Ohio | do 2 fingers | do on the field | do | Doing well 15th day |
| 10 | G. S. | 22 Kentucky | do ring finger | do do | do | do 16th day |
| 11 | J. D. | 54 Indiana | do index finger | Secondary amputation | do | Feverish do |
| 12 | J. M. | 16 Ohio | do do | Primary do | do | Doing well 16th day |
| 13 | D. D. | do | Fingers of right hand destroyed by shell | Secondary do | do | do do |
| 14 | J. M. R. | 4 Iowa | Left hand | None | | do do |
| 15 | L. C. | 16 Ohio | Right hand | Amputation of thumb | do | do do |
| 16 | S. Z. | 114 Ohio | Right index finger | Secondary amputation | do | do do |
| 17 | A. C. | do | do | do | do | do do |
| 18 | N. T. | do | do | do | do | do do |
| 19 | A. S. P. | 30 Iowa | Wound, right hand | None | | do do |
| 20 | H. B. | 16 Ohio | Wound, left hand | do | | do do |
| 21 | C. P. | do | Shell wound, right hand | Index finger amputated | | do do |
| 22 | C. M. | 58 Ohio | Right hand | None | | do do |
| 23 | J. F. | 114 Ohio | do | do | | Doing toler'y well 15th day |
| 24 | W. Z. | do | Fracture of 2 fingers | Secondary amputation | do | Doing well 15th day |
| 25 | G. S. | 16 do | do left ring finger | Amputated | do | do 17th day |
| 26 | M. L. | 7 Mich. Bat. | Flesh wound, left hand | None | | On duty 17th day |

| Case. | Name. | Regiment. | Injury. | Operation. | Anæsthetic. | Remarks. |
|---|---|---|---|---|---|---|
| 27 | J. T. | 54 Indiana | Fracture of all fingers on left hand | Amputated all | Chlor. | Doing well 17th day |
| 28 | J. McG. | 16 Ohio | Articulation of index finger shot out | No operation | | Finger saved |
| 29 | A. L. | 54 Indiana | Shell wound, right hand | do | | Doing well 17th day |
| 30 | P. B. F. | 55 Illinois | Fracture of two fingers | Amputation of 1 | do | do do |
| 31 | A. R. | 29 Missouri | do ring finger, right hand | do | do | do do 18th day |
| 32 | W. C. | 13 Illinois | Shot, left hand | do of hand | | Not doing well 18th day |
| 33 | G. S. | 31 Missouri | do | None | | Died 12th day of tetanus |
| 34 | J.J.H.Y. | 116 Illinois | Compound fracture, 2d metacarpus | Resection of metacarpal; finger afterwards removed | do | |
| 35 | J. B. | 83 Indiana | Compound fracture, left index finger | Amputation on field | None | |
| 36 | A. J. P. | 83 Ohio | do right do | do | do | |
| 37 | J. H. | 55 Illinois | Shot through left hand | None | do | |
| 38 | M. C. | 127 Illinois | Compound fracture, thumb | Primary amputation | Chlor. | |
| 39 | T. T. | 6 Missouri | Shot in left wrist | None | | |
| 40 | T. C. | 54 Ohio | Compound fracture, metacarpus | do | | |
| 41 | G. C. | 116 Illinois | Compound fracture, right index finger | Amputation | do | |
| 42 | J. S. | 6 Missouri | do left thumb | do | do | |
| 43 | F. B. | 116 Illinois | do metacarpus, left hand | Removed fragments | do | |
| 44 | W. C. | 13 Regulars | do left wrist | do | None | |
| 45 | C. C. M. | 22 Kentucky | Sub-luxation of wrist | Reduced | do | Nearly well 7th day |
| 46 | G. C. | 16 Ohio | Shot across little finger | None | | do 10th day |
| 47 | J. V. O. | do | do left index finger | do | | do |
| 48 | J. M. B. | 54 Indiana | do right do | do | | do |
| 49 | H. M. | 120 Ohio | Index finger shot off | Ampntation | Chlor. | Doing well 8th day |
| 50 | J. S. | 16 Ohio | do | do | None | do do |
| 51 | E. H. | 54 Indiana | Flesh wound, hand, slight | None | | do do |
| 52 | G. E. | 16 Ohio | Compound fracture, finger | Amputated | Chlor. | do do |

*Addenda from notes of other Western Battles.*

| | | | | | | |
|---|---|---|---|---|---|---|
| 53 | W. W. | 17 Illinois | Flesh wound, left hand | None | | Recovered |
| 54 | Col. P. | 10 do | Compound fracture, left wrist | do | | do hand saved |

| | | | | | | |
|---|---|---|---|---|---|---|
| 55 | B. B. | 42 do | Compound fractnre, right metacarpus | Excised 1 metacarpal bone | Chlor. | do |
| 56 | Z. W. | 13 Iowa | Flesh wound, right hand | None | | do |
| 57 | —— | 6 Ill. Cav. | Buckshot through hand | do | | do |

With the above should be reckoned about 20 trivial wounds of the hand, received in the fights near Vicksburg, which did not leave the regiment except for one dressing. Total wounds of the hand, 77.

### WOUNDS OF HIP.

| | | | | | | |
|---|---|---|---|---|---|---|
| 1 | E. H. | 49 Indiana | Ball ent'd n'r ant. sup. spin. process of ilium, & travers'd flesh n'r post. sup. spin. process | Ball extracted | Un'kn | Doing well 20th day |
| 2 | J. T. | 120 Ohio | Shell flesh wound left hip and nates | None | | Cannot walk 15th day |
| 3 | A. B. | do | Shell wound right hip and back | do | | Walks 15th day |
| 4 | T. P. | 22 K'ntucky | Flesh wound left hip | do | | Doing well 16th day |
| 5 | J. W. | 120 Ohio | do do | do | | do do |
| 6 | B. F. W. | 13 Illinois | do nates | do | | do do |
| 7 | A. F. | 22 K'ntucky | do left groin | do | | do do |
| 8 | C. S. | do | do left hip | do | | do do |
| 9 | A. K. | 42 Ohio | Ball entered flesh at hip and ran up to arm under the skin | Ball extracted | | do do |
| 10 | E. P. C. | 49 Indiana | Bullet entered above left pubis | Extracted 6 in. right of penis | None | Abscess formed in scrotum, doing well on 17th day |
| 11 | J. B. W. | 16 Ohio | Shell contusion of hip | None | | Left leg entirely paralyzed |
| 12 | J. S. | 54 Indiana | Flesh wound left hip | do | | Doing well 17th day |
| 13 | B. J. C. | 31 Missouri | do do groin | do | | do do |
| 14 | T. D. D. | 30 Iowa | Bullet entered right hip | Cut out middle of thigh | | do 18th day |
| 15 | T. W. G. | 127 Illinois | do left hip and passed out just above knee | None | | do do |
| 16 | J. G. | 29 Missouri | Flesh wound right hip near joint | Not extracted | | do do |
| 17 | J. A. V. | 31 do | do do | None | | do do |
| 18 | W. H. | 29 do | do left hip | do | | do do |
| 19 | A. O. | 13 Illinois | do right groin | Ball not extracted | | Feels well 18th day |
| 20 | P. O. | 6 Missouri | Hip and leg | None | | Doing well 20th day |
| 21 | E. T. O. | 49 Indiana | Shot in left hip | do | | Died 1st day |
| 22 | W. A. K. | 3 Ill. Cav. | Left groin ranging up | do | | do 8th day |

| Case. | Name. | Regiment. | Injury. | Operation. | Anæsthetic. | Remarks. |
|---|---|---|---|---|---|---|
| 23 | M. L. S. | Brig. Gen. | Ball pierc'd ala of ilium & lodg'd in canc'lr tissue of brim of pelvis, viscera not wound'd | Ball pried loose and extracted 10th day | Ether Chlor. | Doing finely 26th day |
| 24 | H. Z. | 83 Indiana | Shell wound of hip, viscera not wounded | None | | |
| 25 | J. A. B. | 54 Ohio | Contusion right hip | do | | |
| 26 | J. W. M. | 13 Regulars | Flesh wound right groin | do | | |
| 27 | W. R. | 6 Missouri | do left hip | Bullet extracted | None | |
| 28 | J. N. | do | Contusion right hip | None | | |

*Addenda from notes of other Western Battles.*

| Case. | Name. | Regiment. | Injury. | Operation. | Anæsthetic. | Remarks. |
|---|---|---|---|---|---|---|
| 29 | J. L. | Unknown | Flesh wound right hip | Bullet extracted | Chlor. | Recovered |
| 30 | H. S. | do | do do also of left leg | None | | do |
| 31 | R. F. | 25 K'ntucky | Bladder pierced and both femurs fractured | do | | Died 19th day |
| 32 | T. S. | Unknown | Flesh wound in groin | do | | Recovered |
| 33 | J. S. B. | 13 Iowa | Ball enter'd cavity of pelvis, viscera unhurt | do | | do |
| 34 | J. B. | 6 Ill. Cav. | Flesh wound, hip | do | | do |

With the above should be reckoned 7 cases of slightly wounded in the hip at the Vicksburg fights, who did not leave their regiments except for one dressing. Total wounds of the hip, 41.

### WOUNDS OF THIGH.

| Case. | Name. | Regiment. | Injury. | Operation. | Anæsthetic. | Remarks. |
|---|---|---|---|---|---|---|
| 1 | W.D.M. | 16 Ohio | Flesh wound, thigh | None | None | |
| 2 | J. T. | 54 Indiana | Right thigh torn off by shell | Amputation on field | Chlor. | Doing very well 15th day |
| 3 | H. C. B. | 16 Ohio | Flesh wound, right thigh | None | | do do |
| 4 | S. B. | 49 Indiana | do do | Ball extracted | None | do do |
| 5 | C.W.G. | 16 Ohio | do do | do 15th day | do | Had erysipelas; pus burrow'd. Better 16th day |
| 6 | J. S. | 54 Indiana | do left thigh | Ball not extracted | | Doing toler'y well 15th day |
| 7 | P. W. | 16 Ohio | do thigh | None | | Feverish 15th day |
| 8 | W. F. | do | do do by shell | do | | On duty do |
| 9 | J. D. | 54 Illinois | do do by bullet | do | | Doing well 15th day |
| 10 | P. C. | 58 Ohio | do right thigh | do | | do do |

| | | | | | | |
|---|---|---|---|---|---|---|
| 11 | M. C. | 4 Iowa | do left thigh | do | | do do |
| 12 | C. G. | 54 Indiana | do right thigh | do | | do do |
| 13 | T. A. | do | do left thigh | do | | do do |
| 14 | J. W. H. | do | Flesh wound, right thigh | None | | Doing well 15th day |
| 15 | S. B. | do | Shell wound, right thigh and knee | do | | do do |
| 16 | J. H. A. | 16 Ohio | Flesh wound, right thigh | do | | do do |
| 17 | T. W. F. | 13 Illinois | do left thigh | Ball extracted | None | do do |
| 18 | W. P. C. | 22 Kentucky | do do | None | | do do |
| 19 | A. S. | do | do do | do | | do do |
| 20 | C. M. | 54 Indiana | do right thigh | do | | do do |
| 21 | R. E. | 16 Ohio | do left thigh | Ball not extracted | | do do |
| 22 | J. W. C. | 49 Indiana | Severe flesh wound of thigh | 2nd'ry hæmor. 7th day from profunda,—tied the vessel; hæmor. again 9th day from femoral, tied that also | Chlor. | do 20th day |
| 23 | G. T. | 29 Missouri | Flesh wound, thigh | None | | do 17th day |
| 24 | A. W. | 58 Ohio | do right thigh | do | | do do |
| 25 | B. A. | 22 Kentucky | do thigh | Cut out bullet | | do do |
| 26 | S. S. | 54 Indiana | do right thigh | Bullet not extracted | | do do |
| 27 | W. W. F. | 16 Ohio | do do | None | | do do |
| 28 | J. C. | 22 Kentucky | do do | do | | do 18th day |
| 29 | M. L. | do | do left thigh | do | | do do |
| 30 | A. D. | 16 Ohio | do thigh | do | | do do |
| 31 | H. H. | do | do left thigh | do | | do do |
| 32 | J. Y | do | do right thigh | do | | do do |
| 33 | J. A. R. | do | do left thigh | do | | do 17th day |
| 34 | J. S. C. | 22 Kentucky | do do | do | | do do |
| 35 | T. S. | 83 Indiana | do thigh | do | | do 18th day |
| 36 | B. S. | 6 Missouri | do do | do | | do do |
| 37 | J. G. M. | 13 Illinois | do do | do | | do do |
| 38 | A. E. B. | do | do left thigh | do | | do do |
| 39 | G. W. W. | —— | do both thighs | do | | do do |
| 40 | C. J. | 8 Missouri | Ball ent'd thigh n'r knee, escap'd from hip | do | | do do |
| 41 | C. M. C. | 6 do | Comp. frac. left thigh & flesh wound of right | Left thigh amputated on field | do | do do |

| Case | Name. | Regiment. | Injury. | Operation. | Anæsthetic. | Remarks. |
|---|---|---|---|---|---|---|
| 42 | L. H. | 6 Missouri | Flesh wound, both thighs | None | | Doing well 18th day |
| 43 | F. T. | 29 do | do right thigh | do | | do do |
| 44 | J. J. Y. | 116 Illinois | do left thigh | do | | do do |
| 45 | C. H. | 13 Regulars | do do | do | | do do |
| 46 | G. H. | 31 Missouri | do thigh | do | | do do |
| 47 | M. F. B. | 13 Illinois | do right thigh | do | | do |
| 48 | J. B. | 25 Iowa | do left thigh | do | | do do |
| 49 | A. R. | 31 Missouri | do do | do | | do do |
| 50 | J. S. | do | do right thigh | do | | do do |
| 51 | G. R. | 6 do | do both thighs and scrotum | do | | do do |
| 52 | S. C. | 4 Iowa | do left thigh | do | | do 20th day |
| 53 | D. L. C. | 31 Missouri | do both thighs | do | | do do |
| 54 | W. McA | 13 Regulars | do left thigh | do | | do do |
| 55 | E. R. | 13 Illinois | do right thigh | do | | do do |
| 56 | A. M. | do | do left thigh | do | | do do |
| 57 | J. W. | 31 Missouri | do do | do | | do do |
| 58 | G. S. | 42 Ohio | do do | do | | Died of typhoid fever 12th day |
| 59 | J. B. S. | 49 Indiana | Compound fracture, thigh | do | | Died 13th day |
| 60 | G. H. | 69 do | Shell wound, both thighs and scrotum | do | | Died of pyæmia 10th day |
| 61 | P. H. | 42 Ohio | Compound fracture, thigh | do | | Died 1st day |
| 62 | J. C. S. | 69 Indiana | do left middle 3rd | do | | Died 10th day |
| 63 | J. C. I. | 114 Ohio | Flesh wound, upper 3rd, thigh, and penis | do | | Died 8th day of erysipelas |
| 64 | J. W. R. | 54 Indiana | Compound fracture, middle 3rd | do | | Died 5th day |
| 65 | P. F. | 57 Ohio | do both femurs, by grape, and left femoral artery cut | do | | Died 1st day |
| 66 | J. C. | 6 Missouri | Flesh wound, thigh and ankle | do | | |
| 67 | M. S. | do | do do | do | | |
| 68 | J. M. | do | Contusion, right thigh | do | | |
| 69 | G. W. | do | Flesh wound, both thighs | Bullet extracted | Chlor. | |
| 70 | J. B. | 22 Kentucky | do thigh | None | | Tendency to gangrene |
| 71 | J. M. C. | 49 Indiana | Shell flesh wound, both thighs,—bad case | do | | |

*Addenda from notes of other Western Battles.*

| | | | | | | |
|---|---|---|---|---|---|---|
| 72 | O. S. | Unknown | Comp. fracture, femur, by cannister-shot | Primary ampu., upper 3rd | Chlor. | Died in 24 hours |
| 73 | Unkno'n | do | do left femur | do middle 3rd | do | Died 3rd day on boat |
| 74 | do | do | do right femur | do upper 3rd | do | Died 4th day do |
| 75 | do | do | do femur,—musket | do lower 3rd | do | Alive 14th day |
| 76 | E. S. S. | 8 Missouri | do leftfemur: artery destroy'd | do upper 3rd | do | Died 5th day |
| 77 | G. B. | do | Compound fracture, middle of femur | Pick'd out fragments, resected ends, put up in splints | Chlor. | Died 4th day |
| 78 | R. C. | 55 Illinois | do do | do do do | do | Died 3rd day |
| 79 | H. H. | 6 Missouri | Bad contusion in front of thigh; muscles reduced to pulp | Crucial incision | | Alive 14th day |
| 80 | S. S. B. | 30 Indiana | Ball enter'd top & front of thigh: flesh wn'd | Enlarged opening to search for bullet | None | Died of gangrene 9th day |
| 81 | J. McC. | 46 Illinois | Compound fracture, upper 3rd femur | Amput. 6th day, at upper 3rd | Chlor. | Died 7th day of shock of operation |
| 82 | H. H. | Unknown | Flesh wound, left thigh | Extracted bullet | None | Recovered |
| 83 | C. K. | 43 Illinois | Compound fracture, femur, upper 3rd | Amputation, upper 3rd | Chlor. | do |
| 84 | D. F. | 48 do | Flesh wound, thigh | Bullet extracted | None | do |
| 85 | F. R. | 12 Iowa | Compound fracture, upper 3rd femur | None | | Alive 35th day; bone exfoli. |
| 86 | Lieut. C. | Unknown | Comp. frac., neck of femur, by ball; lay 20 hours on field, and froze tips of toes | Long splint applied, and adhesive strap extension | | Recovered with shortening; walks about |
| 87 | F. H. | 40 Illinois | Flesh wound, right thigh | None | | Recovered |
| 88 | —— | 70 Ohio | Shot through thigh, wounding femoral artery; secondary hæmor. 10th day | Ligated femoral artery 10th day | | do |

**With these should be reckoned 19 slight cases of injuries to the thigh, received at the fights near Vicksburg, which were returned to their regiments. Total wounds of the thigh, 107.**

WOUNDS OF KNEE.

| | | | | | | |
|---|---|---|---|---|---|---|
| 1 | R. W. | 49 Indiana | Fracture, knee | Ampu. (prim.) lower 3rd thigh | Chlor. | Doing well 20th day |
| 2 | C. G. | 16 Ohio | Flesh wound | None | None | do passably 15th day |
| 3 | D. L. | 54 Indiana | Bad fracture by shell | Amputation of thigh on field | Chlor. | do well do |
| 4 | F. D. | do | Concussion by shell | None | | Joints swollen, walks with crutches 15th day |
| 5 | G. M. W. | do | do do | do | | Well on 16th day |
| 6 | P. H. B. | 49 do | Gun-shot, left knee, flesh | do | | Erysipelas 16th day |

| Case. | Name. | Regiment. | Injury. | Operation. | Anæsthetic. | Remarks. |
|---|---|---|---|---|---|---|
| 7 | P. K. | 69 Indiana | Flesh shell wound | None | | Doing well 16th day |
| 8 | J. A. C. | 54 do | Compound fracture by bullet | Primary amputation of thigh | Chlor. | Flap sloughed, bone resawed 20th day |
| 9 | J. McG. | 6 Missouri | Bullet wound, left knee and foot; flesh wound, right foot and leg | 2ndary amputation 10th day | do | Not doing well 20th day |
| 10 | J. G. | 31 Iowa | Flesh wound, knee; joint not opened | None | | Doing well 18th day |
| 11 | H. R. | 13 Regulars | Shot through right knee | Thigh amputated on field | do | do do |
| 12 | J. B. M. | 127 Illinois | Shell flesh wound inside of knee | None | | do 20th day |
| 13 | J. N. L. | 42 Ohio | Shot through right knee | Primary amputation, thigh | do | Died 6th day |
| 14 | E. F. C. | 54 Indiana | do do | do do | do | Died without reaction 5th day |
| 15 | C. McC. | 6 Missouri | do left knee | do do | do | |
| 16 | W. S. | 13 Regulars | Flesh wound, left knee | None | | |
| 17 | J. H. L. | 42 Ohio | Compound fracture knee | Amputated thigh, lower 3rd | do | Doing well 6th day |
| 18 | E. T. C. | 54 Indiana | do do | do middle 3rd | do | Died 6th day |

*Addenda from notes of other Western Battles.*

| | | | | | | |
|---|---|---|---|---|---|---|
| 19 | J. Z. B. | 46 Illinois | Compound fracture, knee | No operation | | Died 10th day |
| 20 | E. V. | 42 do | do do | Resection of joint | Chlor. | Recovered |

With the above should be reckoned 5 cases of slight flesh wounds of the knee of no importance, and which remained with their regiments. Total wounds of the knee, 25.

WOUNDS OF LEG.

| | | | | | | |
|---|---|---|---|---|---|---|
| 1 | P. C. | 22 Kentucky | Fracture, tibia | Amputation, middle of leg | Chlor. | Doing well 20th day |
| 2 | V. S. | 49 Indiana | do do | Amputated upper 3rd on field | do | do do |
| 3 | A. V. | 42 Ohio | Flesh wound, calf of leg | None | None | Had erysip., d'ng well 15 day |
| 4 | S. B. | 12 do | Shell w'nd, knee, leg & foot all flesh w'nds | do | do | Doing well 19th day |
| 5 | M. H. | 54 Indiana | Wound of calf (flesh) | do | do | do 15th day |
| 6 | O. S. | 22 Kentucky | Flesh wound by shell | do | do | do do |
| 7 | Z. M. | 34 Indiana | do calf of leg | do | do | do do |
| 8 | A. K. | 54 Indiana | do do | do | | do do |
| 9 | J. M. | 16 Ohio | do in front of tibia | do | | do do |

| | | | | | | |
|---|---|---|---|---|---|---|
| 10 | L. E. C. | 42 Ohio | Bayonet wound in front of tibia | None | | Doing well 15th day |
| 11 | V. M. | 29 Missouri | Flesh wound, calf | do | | do do |
| 12 | J. B. | 54 Indiana | do leg | do | | do 16th day |
| 13 | F. W. | 58 Ohio | do do | do | | do do |
| 14 | J. A. B. | 54 Indiana | do left calf | do | | do do |
| 15 | H. S. | 4 Iowa | do do ankle | do | | do do |
| 16 | A. H. | 16 Ohio | Severe flesh wound | do | | do do |
| 17 | B. H. | 29 Missouri | Flesh wound, left calf | do | | do do |
| 18 | H. A. K. | 13 Illinois | do right calf | do | | do do |
| 19 | W. A. M. | do | do do | do | | do do |
| 20 | G. F. | 42 Ohio | do left leg | do | | do do |
| 21 | H. C. | 13 Illinois | do right leg | do | | do do |
| 22 | E. C. | do | do do | do | | do do |
| 23 | L. S. | 22 Kentucky | Compound fracture, left leg | do | | Doing toler'y well 16th day |
| 24 | J. S. | do | Flesh wound, right leg | do | | Doing badly 16th day |
| 25 | W. E. | 54 Indiana | do leg | do | | Doing well 17th day |
| 26 | D. G. | 42 Ohio | Compound fracture, int. malcolus | do | | No improvement 17th day |
| 27 | S. L. W. | 114 do | Foot torn off by shell | Pr. ampu. at lower 3d of leg | Chlor. | Doing well 18th day |
| 28 | T. P. | 54 Indiana | Flesh wound, right calf | None | | do do |
| 29 | M. W. M. | 16 Ohio | do left do | do | | do do |
| 30 | J. D. | 54 Indiana | do do 2 balls | do | | do do |
| 31 | M. K. | 58 Ohio | do calf | do | | do 17th day |
| 32 | J. W. M. | 43 do | Shell comp. fracture, left leg & right foot | pr. ampu. mid. 3d, l. leg, r. meta | do | do do |
| 33 | W. H. | 54 Indiana | Flesh wound, left calf | None | | do do |
| 34 | A. L. | do | Bullet flesh wound, right leg | do | | do do |
| 35 | G. F. | 31 Missouri | Contusion by shell, right leg | do | | Erysipelas 18th day |
| 36 | J. B. | 17 Missouri | Shell wound, left leg | None | | Doing well 18th day |
| 37 | D. B. S. | 116 Illinois | Bullet wound, calf | do | | do do |
| 38 | T. W. S. | 6 Missouri | Compound fracture, leg | do | | do do |
| 39 | L. J. Kerr | do | Flesh wound, leg | do | | do do |
| 40 | H. W. | 13 Illinois | Grape-shot, left calf | do | | do do |
| 41 | P. S. | 17 Missouri | Shell wound, left leg | do | | do do |
| 42 | N. B. | 54 Ohio | Compound fracture, both bones | do | | do 20th day |
| 43 | C. C. | 31 Missouri | Flesh wound, left calf | do | | do do |

| Case. | Name. | Regiment. | Injury. | Operation. | Anæsthetic. | Remarks. |
|---|---|---|---|---|---|---|
| 44 | F. F. | 58 Ohio | Shell flesh wound, right leg | None | | Doing well 20th day |
| 45 | J. W. P. | 22 Kentucky | Compound fracture, left leg | Primary amputation | Chlor. | Died of pyæmia 15th day |
| 46 | A. R. G. | 49 Indiana | Gun-shot, both legs and 1 hand | None | | Died 1st day |
| 47 | J. C. | do | Compound fracture, left leg | Unknown | | do 9th day |
| 48 | E. D. | 54 do | do do | None | | do 11th day |
| 49 | A. R. | do | Shot in both legs and shoulder | do | | do 7th day |
| 50 | W. R. | 58 Ohio | Shot in ankle | do | | do 11th day |
| 51 | C. A. | 114 do | Flesh wound, both legs | do | | do 15th day of tetanus |
| 52 | W. N. | 13 Regulars | do calf | do | | |
| 53 | P. O. | 6 Missouri | do right leg | do | | |
| 54 | J. E. B. | 120 Ohio | do leg | do | | Nearly well 10th day |
| 55 | J. A. P. | 22 Kentucky | Compound fracture, leg | Primary amputation | do | Doing well 6th day |
| 56 | J. T. | 54 Indiana | do do | do | do | do do |
| 57 | G. W. F. | 57 Ohio | do ankle | do leg | do | do do |
| 58 | J. M. M. | 42 do | do right leg | do do | do | do do |
| 59 | R. W. | 49 Indiana | do leg | do do | do | do do |
| 60 | E. L. | 16 Ohio | do left leg | do lower 3d | do | Doing very well 6th day |
| 61 | P. S. | 49 Indiana | do do | do | do | Doing well 6th day |

*Addenda from notes of other Western Battles.*

| Case. | Name. | Regiment. | Injury. | Operation. | Anæsthetic. | Remarks. |
|---|---|---|---|---|---|---|
| 62 | O. R. | Unknown | Leg taken off by cannon-shot | Flap amputation of thigh | Chlor. | Died 4th day,—no reaction |
| 63 | T. S. | do | Flesh wound, right leg | None | | Recovered |
| 64 | J. McD. | 42 Illinois | Compound fracture, left fibula | Resection of fractured part | do | do |
| 65 | E. S. | 40 do | Flesh wound, left leg | None | | do |
| 66 | E. B. | 21 Missouri | do do | do | | do |
| 67 | —— | 6 Ill. Cav. | Buck-shot went thro' leg between bones | Cut out behind the calf | None | do |

To the above should be added 12 slight injuries of the leg, received in the battles near Vicksburg, which did not leave their regiments. Total wounds of the leg, 79.

### WOUNDS OF FOOT.

| | | | | | | |
|---|---|---|---|---|---|---|
| 1 | S. L. | 29 Missouri | Head of meatarsus of great toe | Ball extracted 12th day | | Doing well 19th day |
| 2 | E. L. | 16 Ohio | Fracture, ankle-joint | Ampu. lower 3d leg on field | Chlor. | do 20th day |
| 3 | T. F. W. | 42 do | do second toe | Amputation at base of toe | do | do do |
| 4 | G. W. T. | 57 do | Foot torn off by shell | do lower 3d leg | do | do 15th day |
| 5 | J. W. W. | 49 Indiana | Right os calcis crushed | Ball extracted on field | None | Considerable swelling 15 day |
| 6 | W. H. | 114 Ohio | Flesh wound, 3 toes | None | | Had erysip.—better 15th day |
| 7 | J. S. | 29 Missouri | do sole of foot | do | | Doing well 15th day |
| 8 | E. T. | 22 Kentucky | do left heel | do | | do 16th day |
| 9 | C. S. | 58 Ohio | do do | do | | Wearing shoes 16th day |
| 10 | W. C. F. | 42 do | do left foot | do | | Doing well do |
| 11 | J. H. | 16 do | Gun-shot, left foot | do | | do do |
| 12 | P. U. | 54 do | do 2 toes, left foot | Primary amputation | | do do |
| 13 | A.J.McG | 29 Missouri | Contusion of right foot by round-shot | None | | Very sore do |
| 14 | J. C. | 22 Kentucky | Grape-shot wound, right foot | do | | Doing well do |
| 15 | E. S. | 31 Missouri | Shell wound, left foot | do | | do do |
| 16 | C. H. | 29 do | do do heel | do | | do do |
| 17 | J. L. H. | 114 Ohio | Compound fracture, right tarsus | do | | do do |
| 18 | T. K. | 22 Kentucky | Wound, right foot | do | | Had erysip.—better 16 day |
| 19 | J. G. K. | 7 Mich. Bat. | Flesh wound, left ankle | do | | Doing well 17th day |
| 20 | E. W. | 54 Indiana | Ball passed through left foot | do | | do do |
| 21 | J. E. | 16 Ohio | Compound fracture, great toe | Secondary amputation | | do do |
| 22 | C. A. | 22 Kentucky | Shot through left foot | None | | Erysipelas; doing well 17 day |
| 23 | W. C. | 76 Ohio | do do | do | | Doing well 18th day |
| 24 | J. H. | 54 Indiana | Shot in right foot | do | | do 17th day |
| 25 | W.H.M. | do | Shell wound, outer side of left foot | do | | do do |
| 26 | M. S. | 22 Kentucky | Canister-shot, right foot | do | | do do |
| 27 | J. K. C. | 29 Missouri | Shot across toes | do | | do 18th day |
| 28 | W. B. | 4 Iowa | Shell wound, heel | do | | do do |
| 29 | A. M. | do | Left foot | do | | do do |
| 30 | T. D. | 6 Missouri | Left big toe | Secondary amputation | Chlor. | do do |
| 31 | J. E. | 29 do | Left foot | None | | do do |
| 32 | S. C. D. | 37 do | Flesh wound, foot | do | | do do |
| 33 | S. W. W. | 13 Illinois | Fracture, foot | do | | do do |

| Case. | Name. | Regiment. | Injury. | Operation. | Anæsthetic. | Remarks. |
|---|---|---|---|---|---|---|
| 34 | J. N. | 114 Ohio | Left heel shot | None | | Died of tetanus 12th day |
| 35 | E. H. | 3 Kentucky | Compound fracture, left foot | Primary amputation, leg | Chlor. | Died of typhoid symp. 11 day |
| 36 | T. D. | 6 Missouri | Compound fracture, great toe | None | | |
| 37 | J. B. | 54 Ohio | Slight wound, left foot | do | | |
| 39 | W. J. | 16 Ohio | do ankle | do | | Recovered early |
| 39 | C. J. | do | do do | do | | do |
| 40 | D. McC. | do | Flesh wound, foot | do | | Doing well 8th day |
| 41 | F. C. | do | do do | do | | do do |

*Addenda from notes of other Western Battles.*

| Case. | Name. | Regiment. | Injury. | Operation. | Anæsthetic. | Remarks. |
|---|---|---|---|---|---|---|
| 42 | A. V. L. | 1st Ill. Art. | Comp. frac. of 1st 3 metatarsals, left foot | Extracted ball & fragments | Chlor. | Recovered |
| 43 | J. K. | 40 Illinois | do of os calcis and astragalus | Resection of fractured parts | None | do |
| 44 | J. S. | do | Flesh wound, left foot | None | | do |
| 45 | M. L. | do | Foot crushed with cannon ball | Amputated on the field | Chlor. | do |

To the above should be added 5 cases of trivial wounds, received in the fights near Vicksburg, which remained with their regiments. Total wounds of the foot, 50.

METHOD OF DEDUCTIONS FROM THE ABOVE DATA.

These tables contain a condensed record of 730 wounds. By the arrangements before mentioned, I was able to follow the history of most of these patients for fifteen or twenty days, at the end of which time the question of life or death is usually settled.

In the following pages I shall assume, therefore, that those who at the end of that period seemed to be out of danger, have recovered. The deaths I enter as actually recorded; and the cases which remained critical, or were not heard from, will appear under the head of doubtful. A few errors may thus creep in, but they will not be sufficient to affect our general conclusions.

We will first consider the wounds in relation to the regions of the body affected, noticing the distribution, mortality, and modes of treatment of each species of injury, and subsequently show the conclusions to be drawn from the various surgical operations and their results.

The wounds were distributed through the body in the following proportions:—

| | | |
|---|---|---|
| Wounds of the | Head, | 50 |
| do. | Neck, | 10 |
| do. | Trunk, (not including pelvis,) | 164 |
| do. | Arm, | 69 |
| do. | Elbow, | 14 |
| do. | Fore-arm, | 43 |
| do. | Hand, | 77 |
| do. | Hip, | 41 |
| do. | Thigh, | 107 |
| do. | Knee, | 25 |
| do. | Leg, | 79 |
| do. | Foot, | 50 |
| Total, | | 730 |

*Injuries of the Head.*

I saw great numbers of these in different battles, of whom, I could obtain no record. My recorded cases are 50 in number, which were distributed as follows: flesh wounds and contusions 30, fractures of the face 9, fractures of the cranium 5. The small number of fractures of the cranium results from the following causes: 1st, many wounded in the brain die on the spot, and never appear before the surgeon; 2d, the face lying in front of the cranium, often shields it; 3d, many bullets striking the cranium obliquely, glance off, merely plowing the scalp. Of these 5 fractures, 2 were from bullets penetrating the brain, and 3, from pieces of shell or oblique bullets. They all died without exception; only 1 was trepanned, and he, without benefit. The general result in military surgery is, that gun-shot fractures of the cranium are fatal, and that trepanning is very seldom useful. In penetrating wounds of the brain, the bullet drives before it numerous fragments of bone, hair, clothing, etc., which lodge in the cerebral substance, and occasion hopeless inflammation. A few unrecorded cases of recovery, however, came to my knowledge, and it is worthy of notice that these were, without exception, wounds of the anterior lobe of the brain, which, for some reason seems to sustain injury with less mortality than any other part.

Of the 9 fractures of the face, 5 recovered, 1 died, and 3 remained in a doubtful state. Bullet wounds in the bones of the face are somewhat prone to be followed by secondary hæmorrhage.

Of the 30 flesh wounds, 16 recovered, 4 died, and 10 remained doubtful. Of the entire 50 wounds of the head, of all kinds, 26 recovered, 10 died, and 14 remained uncertain.

### *Wounds of the Neck.*

These were 10 in number, and were all flesh wounds; 6 recovered, and 4 remained in doubt. Wounds of the large vessels, and fractures of the cervical vertebræ, usually die on the field, at once, without coming to the notice of the surgeon.

### *Wounds of the Trunk.*

Under this head I include the shoulder, but reserve the hips for a separate consideration; as thus considered, the wounds of the trunk were 164 in number; 36 penetrated the lungs, 10

pierced the cavity of the abdomen, 31 were flesh or fracture wounds of the shoulder, and 87 were flesh wounds of various regions, or fractures of ribs, not penetrating any cavity.

Of the 36 wounds of the lung, 12 recovered, 18 died, and 6 were uncertain.

Of the 10 wounds penetrating the cavity of the abdomen, 2 were stabs, and 8 gun-shot wounds. The stabbed cases both recovered; but of the 8 bullet-wounds, 6 died, and 2 remained in doubt. There was very little hope of them, however; and they should, probably, all be reckoned as dead. With very few exceptions, bullet wounds into the abdominal cavity are all fatal. It may be a question worthy of serious thought, in view of the hopelessness of our present practice, whether we ought not to cut boldly into the abdominal cavity, wash out the filth, and bringing the wounded intestine to the surface, endeavor to produce an artificial anus.

Of the wounds of the shoulder, 31 in number: 20 recovered, 2 died, and 11 remained in doubt.

The 87 superficial wounds of the trunk all recovered.

Of the total number of those wounded in the trunk and shoulder, 20 died, 142 recovered, and 2 were doubtful.

Wounds of the head, neck, and trunk, from their nature, seldom admit of much surgical assistance; taken as one class, they present a mortality of about 20 or 30 per cent; which may be somewhat diminished by good care, or horribly increased by bad air in a crowded hospital; but can be little affected by operative measures, except in a few instances.

### *Wounds of the Arm.*

The very opposite is true, however, of the wounds of the extremities; here the skill and sound judgment of the operator are of immense value, and the correctness or error of his measures will produce vast changes in the ratio between mortality and recovery.

Of wounds of the arm, my records show 69 cases, of which, 28 were compound fractures of the humerus, and 41 were flesh wounds. The flesh wounds all recovered; of the factures, 21

recovered, 4 died, and 3 were in doubt. In 6 of the fractured cases, the shoulder-joint was resected; of which, 5 recovered, and 1 died. In 6 others, amputation was performed at the shoulder-joint; of which, 4 recovered, and 2 died. In 8 cases, amputation of the arm was performed; of which, 7 recovered, and 1 is unknown. In 8 cases, no operation was performed, and the fracture was treated with splints; of these, 7 recovered, and 1 died.

The ratio of mortality in all the gun-shot fractures of the humerus is 1 in 7. The question of the grounds of choice, between resections and amputations of the extremities, will be discussed below, under the head of operations.

*Wounds of the Elbow.*

Of these, 4 were flesh wounds, of which, 2 recovered, and 2 are unknown; 10 cases were compound fractures of the joint, of which, 7 recovered, 1 died, and 2 remained undecided. In 4 of the cases, resection of the joint was performed, of which, 3 recovered, and 1 died. In 3 cases, amputation of the arm was resorted to, of which, 2 recovered, and 1 was not decided. In 3 cases of less severity, no operation was performed, and all recovered.

The total number of wounded in the elbow was 14; of whom, 9 recovered, one died, and 4 remained doubtful.

*Wounds of the Fore-arm.*

Of these, 27 were flesh wounds, and 16 were compound fractures. Of the flesh wounds 22 recovered, and 5 were doubtful. Of the compound fractures, 10 recovered, and 6 remained in doubt.

In 4 of the cases, amputation was performed, and all of them recovered; no death, therefore, was observed from wounds of the fore-arm.

*Wounds of the Hand.*

Of these, 38 were flesh wounds, of which, 37 recovered, and 1 died; 25 cases were fractures of the phalanges, of which, 18 recovered, and 7 are unknown; 9 cases were fractures of the metacarpals, of which, 4 recovered, and 5 are unknown; 5 cases were fractures of the wrist, of which, 3 recovered, and 2 are doubtful. 24 fingers were amputated, of which cases, 19 recovered, and 5 were not heard from. One amputation was performed through the metacarpals,—result unknown. One shot across the metacarpals, was very unjustifiably treated by amputation of the fore-arm four inches above the injury; the patient recovered.

Total wounds of the hand 77; known mortality 1.

*Wounds of the Pelvic Region.*

39 flesh wounds of this region occurred, of which, 30 recovered, 2 died, and 7 were undecided; 1 of the 2 cases which died, was wounded in the bladder, and the other perished of secondary hæmorrhage and general exhaustion, from the bad air of an overcrowded boat.

Only 2 cases of fracture of the pelvis were brought to my notice, both of which recovered; the viscera were not wounded in either. Total wounds of the pelvic region 41.

*Wounds of the Thigh.*

This is a most important division of the field of military surgery, and from it spring some of the most trying and difficult questions which are ever laid before the operator for decision. The discussion of these questions will be given below, under the head of operations.

The total number of wounds of the thigh was 107, of which, 89 were flesh wounds, and 18 were compound fractures. Of the 89 flesh wounds, 75 recovered, 3 died, and 11 were doubtful; of the 18 fractures, 5 recovered, 12 died, and 1 was doubtful; 5 of the fractured cases were amputated at the upper third, of which, 1 recovered, and 4 died; 3 were amputated at the middle third, of which, 2 recovered, and 1 died; 1 was amputated at the lower third, and recovered; 2 cases were treated by resecting the fractured portions in the continuity of the shaft, both of these died; 8 cases were treated without operative interference, by simply employing splints, position, and such incisions as were necessary to evacuate pus, of these, 2 recovered, and 6

died. The 2 which recovered were both shot in the cancellar tissue of the neck or trochanter, where my operation must necessarily have been amputation at the hip, or excision of the head of the bone; 1 of them lay twenty hours on the field, in very raw and cold weather. It would seem that shots through the cancellar tissue, at the superior fifth of the femur, are much less dangerous than those in the compact bone of the shaft below; the reason is, that when a ball bores its way through spongy bone, it produces only a moderate amount of shattering, owing to the yielding character of that tissue; but the impact of a minnie bullet upon the brittle ivory of the shaft, shatters it for several inches, and disperses the fragments with the force of an explosion among all the surrounding tissues, producing immense disorganization. These cases nearly all die within the first five days, no matter what treatment is adopted.

### *Wounds of the Knee.*

There were 26 wounds of the region of the knee, of these, 14 were flesh wounds, and 12 were compound fractures; 12 of the flesh wounds recovered, none died, and 2 remained doubtful. Of the 12 compound fractures, 5 recovered, 4 died, and 3 remained doubtful; 10 of these fractures were treated by amputation at the lower third of the thigh, of which, 6 recovered, 3 died, and 1 remained in doubt; 1 case was treated by resection of the knee-joint, and recovered; 1 was treated without any operation, and died. In this connection, it may be remarked that I observed a considerable number of cases of gun-shot fractures of the knee at the battle of Shiloh, very injudiciously treated as ordinary fractures, without any operation; as I could obtain no record of the cases, I have not entered them in the tables, but I never knew one to recover. Let any young surgeon, who is reluctant to sacrifice the limb or joint in these cases, take the trouble to dissect two or three of them, and he will see at once why they all die, unless they are amputated or resected. The bullet disorganizes the interior of the joint in a most surprising manner, filling it with five hundred fragments of bone and cartilage and putting it in a condition from which no human frame can recover without operative help.

*Wounds of the Leg.*

These were 79 in number, of which, 56 were flesh wounds, and 23 were fractures. Of the 56 flesh wounds, 51 recovered, 1 died, and 4 were undecided; of the 23 cases of fracture, 14 recovered, 7 died, and 2 are unknown; 12 of the fractures were treated by amputation of the leg, of which, 11 recovered, and 1 died; 1 was treated by amputation of the lower third of the thigh, and recovered; in 1 case, a portion of the bone was resected, which also recovered; 8 cases were treated by splints, without any operation, of these, 2 recovered, 4 died, and 2 remained doubtful.

*Wounds of the Foot.*

These were 50 in number; 31 were flesh wounds, and all recovered; 4 were fractures of the phalanges, and all recovered; 6 were fractures of the metatarsus, of which cases, 4 recovered, 1 died, and 1 is unknown; 9 were fractures of the tarsus, of which, 7 recovered, 1 died, and 1 remained doubtful; amputation of the toes was performed in 4 cases, which all recovered. No amputation through the metatarsus occurred; one amputation through the tarsus was performed, and the patient recovered. In 4 cases the leg was amputated, of which, 3 recovered, and 1 died. A portion of the tarsus was resected in 1 case, which recovered.

*Predominance of wounds on the Right Side of the Body.*

In western warfare, the constant occurrence of battles in the forest, gives predominance to the operations of skirmishers, who deliver their fire usually from the right hand side of the trees that shelter them; in consequence of this, the right hand, arm, and shoulder, and the right thigh, knee, and leg, receive many more wounds than the left.

*Discussion of the Operations.*

The operations in these cases were, for the most part, executed by well educated and skilful men, so that there was little occasion to criticise them. In respect to the mode of their performance, they will compare favorably with similar operations in any other army. There were some errors of judgment, respecting the kinds of treatment to be decided upon, but not more than was to be expected.

The following tables show the number and locality of the operations:—

*Amputations.*

| | Recover'd. | Died. | Doubtful. | Total. |
|---|---|---|---|---|
| Amputations at the shoulder-joint, | 4 | 2 | | 6 |
| do. of the arm, | 9 | | 2 | 11 |
| do. do. fore-arm, | 5 | | | 5 |
| do. do. hand, | 1 | | | 1 |
| do. do. fingers, | 19 | | 5 | 24 |
| do. do. thigh, upper third, | 1 | 4 | | 5 |
| do. do. do. middle do. | 2 | 2 | | 4 |
| do. do. do. lower do. | 7 | 3 | 1 | 11 |
| do. do. leg, | 14 | 2 | | 16 |
| do. do. foot, | 1 | | | 1 |
| do. do. toes, | 4 | | | 4 |
| Total, | 67 | 13 | 8 | 88 |

No case occurred in which we felt justified in amputating at the hip-joint.

*Resections.*

| | Recover'd. | Died. | Doubtful. | Total. |
|---|---|---|---|---|
| Shoulder-joint, | 5 | 1 | | 6 |
| Elbow-joint, | 3 | 1 | | 4 |
| Parts of hand, | 1 | | 1 | 2 |
| do. shaft of femur, | | 2 | | 2 |
| Knee-joint, | 1 | | | 1 |
| Parts of fibula, | 1 | | | 1 |
| do. foot, | 1 | | | 1 |
| Total, | 12 | 4 | 1 | 18 |

*Ligations of Arteries.*

(Generally for secondary hæmorrhage.)

| | Recover'd. | Died. | Total. |
|---|---|---|---|
| Sub-clavian artery, | 1 | | 1 |
| Sub-scapular do. | 1 | | 1 |
| Facial do. | 1 | | 1 |
| Axillary do. | | 1 | 1 |
| Profunda femoris artery, | 1 | | 1 |
| Femoral artery, | 2 | 1 | 3 |
| Total, | 6 | 2 | 8 |

In reviewing these tables, it is a matter of profound regret, that among some thousands of wounded, who, in different battles have been under the care of myself and others, we were able to trace out the results of so few cases; still, the careful observation of the facts here recorded, combined with statistics from other sources, will help to set at rest the most prominent of the disputed questions of military surgery.

The practical questions before the military operator, are mainly the following:—

1. What cases require amputation?
2. What cases require resection?
3. What cases should be treated without operative interference?
4. What variations from accepted rules must be made, in view of special military exigencies.

First then:—

*What cases require amputation?*—The rule is now well established, that the military surgeon may go almost all lengths in his efforts to preserve superior extremities; but that in the inferior, amputation must be very extensively practiced.

*Amputation of the shoulder-joint.*—This is only required in cases where an arm has been torn off by a cannon-shot, or otherwise so hopelessly disorganized as to render mortification of the whole limb inevitable. If the head of the humerus is shattered, resection should be preferred. In my experience, as shown in the above tables, amputations at the shoulder have had a mortality of one in three, while resections of the joint only showed a loss of one in six.

In the Schleswick, Holstein, campaign, ESMARCH gives the results of 19 resections of the shoulder, of which, 12 recovered, and 7 died. GUTHRIE quotes 44 cases of amputation at the shoulder-joint, in the British Wars with Napoleon, of which, 17 died. Combining all these statistics, we find the following results:—

| | Total number. | Recover'd. | Died. | Per cent of deaths. |
|---|---|---|---|---|
| Amputations at shoulder, | 50 | 31 | 19 | 38 |
| Resections of do. | 25 | 17 | 8 | 32 |

Showing an advantage of 6 per cent in favor of resections.

In addition to the diminished risk, the great value of the preserved limb is to be taken into account. After resection, the use of the elbow and hand is perfect; and some soldiers have even returned to duty as soon as the cure was perfected. In case of doubt whether an arm can be saved, time should be taken to watch the progress of the patient before deciding, for, although primary operations are preferable, yet the secondary ones are very well borne; and it is a man's duty to risk his life to some degree, for so important a member as a superior extremity. GUTHRIE fully sanctions the same opinion, when he affirms that amputations of the superior extremity should not be primary, unless the impossibility of saving the limb is obvious.

Sabre cuts and bullet wounds, simply opening the shoulder-joint, without serious comminution of the bone, do not render either resection or amputation necessary, as the patient recovers with anchylosis, in the majority of instances. If, however, the head of the humerus is badly comminuted, an operation of some kind is absolutely required, as the mortality in cases treated simply with splints, is found to be over 60 per cent.

*Amputations of the arm.*—These should only be performed when there is no possibility of preserving the limb. Amputations for bad fractures of the humerus, or for shattered elbows, while there is still a good pulse at the wrist, are no longer justified by any respectable authority. It is often astonishing to inexperienced surgeons to see from what terrific injuries a wounded arm will recover itself. If the bone is shattered, the artery cut, and the anastomotic vessels also so extensively destroyed, that circulation in the limb ceases, amputation should be immediately resorted to. If, however, circulation continues in some measure below the injury, the loose fragments of bone should be picked out, and the limb dressed as for other compound fractures.

The mortality after amputations of the arm is but slight; of 11 cases in my tables, not one died. Of 72 cases mentioned by GUTHRIE, only 17 died. Combining these statistics, we have the following result:—

| | Total number. | Recover'd. | Died. | Per cent of deaths. |
|---|---|---|---|---|
| Amputations of the arm, | 83 | 66 | 17 | 20½ |

*Amputations in the fore-arm and hand.*—As we recede from the body, both operations and injuries become less fatal. All the cases of amputation of the fore-arm and hand, of which I could obtain the results, recovered. The few who die, succumb not to the operation, but to the secondary effects of the deadly air of overcrowded hospitals. In every case where required, the amputation may be resorted to without fear; but it should be borne in mind that the fore-arm and hand recover from the most frightful looking wounds with surprising ease, and that every inch which can be preserved is of priceless value to the patient. In a mangled hand, almost every part which is not torn off, may be preserved, and should be, generally, retained. I make these remarks, because I have observed that inexperienced surgeons will often be moved by the ghastly appearance of a fractured and lacerated hand, to undertake very unjustifiable amputations.

*Amputations at the hip-joint.*—No case of this fell under my notice, as we all adopted the principle, that it was an operation which can scarcely ever be justified.

*Amputations of the thigh.*—In this part of the body, we reverse the rules applied to the superior extremity. Instead of going all lengths to save the member, we incline more decidedly to prompt and resolute amputation on the field. Secondary amputations of the thigh are usually fatal, therefore, the decision of the surgeon must be made up on the spot, from the appearance of the case, and resolutely carried out. My records show 20 amputations of the thigh, of which, 9 died, 10 recovered, and 1 remained doubtful, being a mortality of about 45 per cent. It is of the utmost importance here to observe the difference of mortality between the upper and lower parts of the thigh, because, on this difference are based life and death decisions. The following table illustrates it:—

| | Total cases. | Recover'd. | Died. | Doubtful. | Per cent of deaths. |
|---|---|---|---|---|---|
| Amputated upper 3d of thigh, | 5 | 1 | 4 | | 80 |
| do. midlle do. | 4 | 2 | 2 | | 50 |
| do. lower do. | 11 | 7 | 3 | 1 | 27 |

Showing plainly that "every inch by which this operation approaches the body, increases its danger."

According to LONGMORE'S statistics, a similar percentage was observable in the Crimean Campaign, as is shown by the following table:—

| | Per cent of deaths. |
|---|---|
| Amputation, upper third, in Crimean War, . . . . . . . | 87 |
| do. middle do. do. . . . . . . | 60 |
| do. lower do. do. . . . . . . | 57 |

These figures show a more favorable result in our army than in the British, by an average of about 20 per cent. Combining the two tables, we have approximately the following:—

| | Per cent of deaths. |
|---|---|
| Mortality of amputation at upper third, . . . . . . | $83\frac{1}{2}$ |
| do. do. middle do. . . . . . . | 55 |
| do. do. lower do. . . . . . . | 42 |

The obvious deduction of which, is that the amputation should be made as far from the body as the nature of the injury will possibly permit. Such being the frightful mortality of amputations of the thigh, I tried in two cases to produce a better result, by resecting the ragged ends of the broken femur, and then treating it as for compound fracture. Both these cases died within the fifth day. The same experiment was tried on the Potomac, by Eastern surgeons, and also in the Crimea, and always with the same result,—every case proving fatal.

Still, other experiments have been made, by treating the case simply as a fracture, without any other operation than an incision to evacuate the pus. STROMEYER quotes 4 cases of recovery. My tables show 8 cases treated in this manner, of which, 2 recovered, and 6 died. These cases were mostly fractures above the middle; hence the mortality of 75 per cent is not greater than would have followed amputation in the same place. In Europe, after the battle of Toulouse, this mode was tried on 43 of the most favorable cases, with a mortality of about 60 per cent, which, on the whole, is not much worse than the results of amputation, which, in nearly all fractures of the femur, must be as high as the middle, and has a mortality of 55 per cent.

A careful, and very deliberate examination of this whole matter, has settled in my mind the following conclusions:—

1st.—A very large portion of the cases with badly comminuted femurs, will die within five days,—under all treatments, alike. There is no perfect reaction.

2d.—Shots through the spongy tissue of the trochanter and neck of the femur, are less fatal than those through the compact tissue of the shaft. This is contrary to STROMEYER's opinion; but it is nevertheless true. The splintering of the bone, and consequent injury of soft parts, is far less in this spongy part than in the ivory-like shaft below. These cases of fractured neck, require neither amputation nor resection of the head of the femur; a large part of them will recover with simple extension-splints, and in some cases, incisions to evacuate pus; whereas, amputations and military excisions at the hip-joint may be practically said to be all fatal. I know of 2 cases of this fracture which recovered without difficulty in straight splints.

3d.—Amputations above the middle of the femur should only be resorted to in desperate circumstances, where the limb below is either torn off, or is so injured that it has but little prospect of escaping mortification. If the circulation and innervation are good below, a free incision should be made down to the comminuted bone, and the limb be dressed with a straight splint and adhesive-strap extension-bands. The case is a desperate one, but I am confident that this treatment will save more lives than amputation above the middle.

4th.—If amputation can be made below the middle of the thigh, it should be promptly performed, for all severe compound fractures of the lower half of the shaft of the femur, and all gun-shot fractures of the knee-joint. By this treatment, about

75 per cent of the patients may be saved; but if attempts are made to save the limb, almost every man will die. At the battle of Shiloh, a large number of cases were treated with this false conservatism, and many lives sacrificed in consequence. If any young surgeon feels reluctant to sacrifice a fair and plump thigh, for a mere little bullet hole of very harmless appearance in the knee, I advise him first to amputate, and afterwards to dissect the limb; he will find within the joint a horrible disorganization, such as no man can reasonably hope to survive, without operative assistance.

*Amputations of the Leg.*—These may be resorted to whenever a useful limb cannot be preserved, as the operation is not excessively dangerous. If, however, the circulation in the foot continues, and a chance of future usefulness of the member presents itself, conservative surgery should be practiced; because the danger of postponing or omitting amputation is not great, even though the foot should mortify. One hint may serve to guard young surgeons against a natural error: when a bullet traverses through the tibia from before, backwards, the front opening in the skin is small; but the fragments of the bone are driven back among the tissues of the calf, producing more danger of mortification than the first glance indicates. On the other hand, if the ball has traversed from behind, forwards, it drives all the splinters outward through the skin in front, doing less real injury than in the former case, but still tearing open the skin, and everting the flesh over an area of two or three inches in diameter. The wound looks so hideous, that it is not uncommon for the inexperienced operator to be moved by it to cut off the better limb and save the worse.

*Amputations of the foot.*—These may be decided upon and executed by the same rules as in civil surgery.

### *Resections.*

*Resection of the shoulder-joint.*—The grounds of choice between this and amputation have already been discussed under the head of "Amputations at the shoulder." It is to be preferred, in proper cases, both for its superior safety, and because it saves a most important limb.

*Resection of the elbow.*—My tables show 4 cases of this resection, of which, 3 recovered, and 1 died. ESMARCH quotes 40 cases, of which, 6 died. Combining the two sets, we have this table:—

| | Number of cases. | Recover'd. | Died. | Per cent of deaths. |
|---|---|---|---|---|
| Resection of elbow-joint, | 44 | 37 | 7 | 16 |
| Amputation of arm, | 83 | 66 | 17 | 20½ |

Showing an apparent advantage of 4½ per cent in favor of resection. As amputation, however, was often for severer injuries than those which required resection, it will, probably, be fair to assume that in injuries which admit of the choice, the risks of the two operations are about equal; but as resection preserves, and amputation loses the hand, the choice is unquestionably for the former. I, therefore, advise resection for all comminuted gun-shot fractures of the elbow-joint, in which the preservation of the hand is not hopeless from gangrene.

*Resections of parts of the hand.*—These should be governed by the same rules as in civil practice.

*Resections of the knee-joint.*—The great mortality of amputations of the thigh, has caused this operation to be proposed as a substitute in cases of bullet wounds of the knee. My tables show only one case, and that recovered. From all sources, European and American, I am able to collect accounts of only 8 cases in military practice, of which, 2 recovered, and 6 died; a mortality of 66 per cent, which is 24 per cent worse than that of amputations at the lower third of the thigh. More extensive statistics, however, are needed to settle its true value. At present I advise, both from my own observations and careful review of the opinions of other surgeons, that in case good air, and freedom from motion can be had for the patient, resection of the knee may be preferred; but, if he must be transported far in an ambulance, or put in a crowded hospital, where there is less than 1200 cubit feet of fresh air for each patient, resection will prove fatal. Amputation should then be at once performed, for delay with a view to secondary resection is not to be thought of.

*Resections in the leg and foot.*—These are well-borne, and follow the same rules as in civil practice.

*Anesthetics.*

Chloroform was freely used in most of the painful operations. A mixture of chloroform and ether was used in one case. Ether alone was not used, to my knowledge, in any case. Chloroform was administered in 113 cases, without any accident.

*Diseases of overcrowded Hospitals.*

There is a class of deadly complications following the injuries of patients after nearly every large battle, which, are almost solely the product of overcrowding and bad air. These are the following:—

Erysipelas,
Diffusive phlebitis,
Pyæmia,
Hospital gangrene.

About 10 or 15 per cent of the deaths in military surgery are from these causes, and I regret to say, that in many instances these dead are slain by the surgeon, whose stupid ingenuity was all expended in procuring beds in warm and close quarters, where the patients poison each others' blood, instead of having free air where they may breathe and live.

After the battles at Vicksburg, the wounded were put upon three steamboats; but by accident were not evenly distributed. It thus happened that the small steamer "Von Phul" received over 300 cases, while the large boat "City of Memphis" had only 120. This arrangement, owing to military movements, could not be changed under about ten days; the results were disastrous,—but yet instructive. About the fifth day, I was ordered to take command of the "Von Phul." Going on board, I found about half the patients crowded into the cabins and state-rooms, where they had, by measurement, only 250 cubic feet of air per man, when they should have had not less than 1200 feet each. The windows and doors were mostly closed, and an overpowering stench of putrifying pus pervaded everything. Erysipelas and pyæmia had already commenced; and secondary hæmorrhage and gangrene were quite common. The patients were rapidly dying, and every wound, without exception, presented a bad, unhealthy aspect. Meanwhile, the rest of the patients who occupied the open decks, outside, were doing well. Almost every death was in the cabin. I immediately opened all the windows and doors, and ordered a large portion of the wounded to be carried out and laid upon the decks. In this way, the evil was mitigated, but much mischief was already done. By the tenth day, we had lost 45 patients, or one-eighth of the entire number. Meanwhile, the "City of Memphis," with her small numbers, and vast airy cabins, had only lost 1 patient in 20. On the tenth day, the military commanders committed the enormous blunder of ordering all the wounded of the three boats to be concentrated upon the "City of Memphis." This, however, being the largest boat in the fleet, did not prove so bad as might have been feared; but it was a most unwise arrangement, and would have cost some further lives, but for the great care exercised over ventilation, by Dr. TURNER and his assistant, Dr. WITT.

I have observed with pain, that partly by military necessity, and partly by ignorance of ventilation displayed by surgeons, this error of overcrowding is repeated after almost every large battle, and perpetuated in most of our large General Hospitals.

If the weather is not so inclement as to endanger death from cold, I have no doubt that by far the best plan is to keep the patients dispersed for two or three weeks in open tents and booths in the field; although, in this way they have less comfortable beds, and coarser food than in Post Hospitals, they get fresh air, and with that they often survive the most desperate wounds.

It is often remarked, that men wounded in occasional skirmishes, where they are kept with the Regimental Hospital in the field, seldom have erysipelas or pyæmia, and recover from their injuries far more readily than those sent away to large, square, six story buildings, like the Overton Hospital in Memphis, where overcrowding is frequently unavoidable, and perfect ventilation an impossibility.

The results of my observations in the army, under this head, may be summed up, therefore, in one sentence:—Let the military surgeon see that he gets fresh air for his men in preference to food, warmth, or shelter.

Men will lie in snow, on wet ground, or under open sheds, and do well on bacon and hard bread; but, in close hospitals they will die, though they have all the luxuries of the world around them.

